# Plasticity, Variability and Age in Second Language Acquisition and Bilingualism

**DOI:** 10.3389/fpsyg.2018.00081

**Published:** 2018-03-12

**Authors:** David Birdsong

**Affiliations:** Department of French and Italian, The University of Texas at Austin, Austin, TX, United States

**Keywords:** second language acquisition, bilingualism, plasticity and learning, variability, age factors, individual differences, critical period, dominance

## Abstract

Much of what is known about the outcome of second language acquisition and bilingualism can be summarized in terms of inter-individual variability, plasticity and age. The present review looks at variability and plasticity with respect to their underlying sources, and at age as a modulating factor in variability and plasticity. In this context we consider critical period effects vs. bilingualism effects, early and late bilingualism, nativelike and non-nativelike L2 attainment, cognitive aging, individual differences in learning, and linguistic dominance in bilingualism. Non-uniformity is an inherent characteristic of both early and late bilingualism. This review shows how plasticity and age connect with biological and experiential sources of variability, and underscores the value of research that reveals and explains variability. In these ways the review suggests how plasticity, variability and age conspire to frame fundamental research issues in L2 acquisition and bilingualism, and provides points of reference for discussion of the present *Frontiers in Psychology* Research Topic.

## Introduction

This review article examines a range of features of second-language (L2) acquisition and bilingualism from the intersecting perspectives of plasticity, variability and age. In the simplest terms, for the L2 context plasticity is a property of the neuro-cognitive mechanisms, structures and systems that enable and constrain L2 learning. Variability in L2 attainment at the individual level is conditioned by factors that may be experiential, biological, intellectual, linguistic, conative, educational, and identificational in nature.

Both variability and plasticity are modulated by the age when L2 learning begins [Age of Acquisition (AoA); see below]. Both main and interactive AoA effects on plasticity have been attributed to neurological maturation, to neurochemical and hormonal fluctuations, to decrements of cognitive function over time, to decreases in regional brain volume, to the degree of first- language (L1) entrenchment at the initial state of L2 acquisition, and to the relative use and maintenance of the L1 vs. the L2 (e.g., Birdsong, 2006; Muñoz and Singleton, 2011). AoA may also indirectly condition learner variables such as the extent to which an individual is motivated to acquire an L2 to high levels of proficiency, to engage in the L2 culture, and to identify with L2 speakers (e.g., Dörnyei and Skehan, 2003; Moyer, 2014).

A comprehensive synthesis of relevant research is beyond the scope of this article. Rather, by use of selected examples, the goal is to expose the essential nature of L2 acquisition and bilingualism from the perspectives of age, plasticity and variability. From these perspectives, we can conceive of linguistic attainment in terms of factors that make L2 learners and bilinguals different from monolinguals, and perhaps get a sense of why these differences are not necessarily deficiencies.

After a brief orientation to the developmental neurobiology of age and plasticity in language learning, I consider the evidence for critical periods in L2 acquisition, taking into account the shape of the function that relates AoA to attainment and the (im)probability of nativelike attainment. The next section offers two illustrations of sources of variation in learning outcomes, which include not only AoA, but also the particular linguistic features under investigation and individuals’ cognitive styles and capacities. In the next section I examine possible sources of greater heterogeneity of attainment of L2 morphosyntax with increasing AoA. This is followed by consideration of inter-individual differences, first with respect to exceptional L2 learners and polyglots, then in terms of neurogenetically based talent and trainability, then as a function of idiosyncratic construction of categories for representing linguistic form. In the final section I look at several ways in which linguistic dominance instantiates concerns about plasticity and age, and at how the dominance factor can account for variability in pronunciation and language learning among individual bilinguals.

The works reviewed here converge on the conclusion that studying non-uniformity in language learning outcomes is not so much about sifting through noise and scatter in the data, as it is about illuminating an inherent characteristic of both early and late language acquisition. To this end, it is important to show how plasticity and age connect with biological and experiential sources of variability, and to orient research questions in ways that expose and exploit variability.

A basic motivation of this review is to provide points of reference and theoretical and empirical foundations for readers of the other contributions to the present *Frontiers in Psychology* Research Topic. In so doing I hope to give a sense of how plasticity, variability and age conspire to frame fundamental research issues in L2 acquisition and bilingualism.

### Notes on Terminology and Concepts

In this review, the relationship between age and L2 attainment will be considered with respect to the time at which learning of the L2 begins, be it from birth or at any time thereafter. The term AoA refers to the age at which L2 learning begins in earnest and continues with little or no interruption, most often in immersion contexts such as immigration, but not to limited acquaintance with the L2 that takes place in on trips or in the foreign-language classroom. Note that some studies use the terms Age of Exposure, Age of Immersion or Age of Arrival.

The point at which L2 learning begins is conceptualized as the *initial state* of L2 acquisition: the sum of an individual’s cognitive, neurological, and linguistic development, along with motivational, identificational, attitudinal and experiential characteristics. Since this cluster of features is difficult to quantify, AoA is taken to be a proxy for the L2 acquisition initial state. In this sense, L2 AoA is understood not as the “age factor” but rather as a “meta-variable” (Flege, 1999). As a predictor variable in statistical analyses, AoA can be applied to both bilingual (simultaneous or sequential) development in childhood, and to immersion and immigration contexts later in life.

In this review, *bilingualism* is understood to mean routine use of two languages, at whatever level of proficiency in either language. Bilinguals who are immigrants or migrant workers may have acquired their two languages naturalistically only, or they may have had some classroom experience followed by immersion and frequent use. Over the past decade, a disciplinary “bilingual turn” (Ortega, 2009; May, 2014) in language studies recognizes that “L2 learners” and “bilinguals” are not always distinct populations. Obviously, this conflation does not apply to training studies where, for example, participants are taught an artificial language, Mandarin tone contrasts, or the /r/-/l/ distinction in English. Nevertheless, AoA is commonly employed as a predictive factor for learning outcomes in training studies.

In this contribution *critical period* is intended as a generic term that subsumes *sensitive period*. The latter term is sometimes used in contexts of relatively mild maturational effects; at other times it is only meant to suggest heightened receptivity (sensitivity) to relevant environmental stimuli. Both terms refer to finite developmental spans, which may range from birth up to adulthood. In some studies, critical period is taken to mean just the peak period of plasticity or receptiveness of the learning system; in other studies (including the present one), the critical period begins when plasticity starts to increase above baseline and continues until plasticity has leveled out. Maturational effects are thought to take place within, but not beyond, the critical period. For this reason one distinguishes maturational effects from general age effects over the lifespan and, similarly, from AoA effects. For a synopsis of the literature on critical periods for language and other domains, see Birdsong (2017).

Finally, for the purposes of this paper, *learning* and *acquisition* will be used interchangeably. (In some studies, the former term is reserved for formal instructional contexts.)

## Plasticity, Variability and Age: Developmental Neurobiology and Behavioral Outcomes

The notion of plasticity with respect to adult language acquisition is often traced back to Penfield and Roberts (1959, p. 240), who argue that for recovery from aphasia the adult brain is “inferior” while the child brain is “plastic,” that is, more likely to regain language function. Also seminal in this regard are passing remarks by Lenneberg (1967, p. 176), who links L2 learning difficulties in adulthood with hemispheric functional specialization and declines in plasticity that constrain primary language acquisition.

More recent researchers have put forth other neurobiological explanations for plasticity deficits over age. For example, on a “use it then lose” it model, after adolescence the circuitry that is required for language learning is dismantled because in adulthood there remains no selection pressure on humans to keep learning languages and the metabolically greedy neural systems that subserve language learning (Hurford, 1991; Pinker, 1994). Another proposed neurobiological culprit is maturationally regulated myelination in the circuitry that underlies language learning. On this view, myelination insulates axons for efficient transmission of electrical impulses, but does so at the cost of reducing the synaptic plasticity required for new learning (Long, 1990; Pulvermüller and Schumann, 1994). Declines in nigrostriatal dopamine over age are implicated in decrements of cognitive abilities such as attention, sequencing, and suppression of competing information; these domain-general capacities are put to use in online L2 processing (Lee, 2004; Wong et al., 2012; see below). The regulation of plasticity takes place within a critical period, “a bounded maturational span during which experiential factors interact with biological mechanisms to determine neurocognitive and behavioral outcomes” (Birdsong, 2017).

**FIGURE 1:**
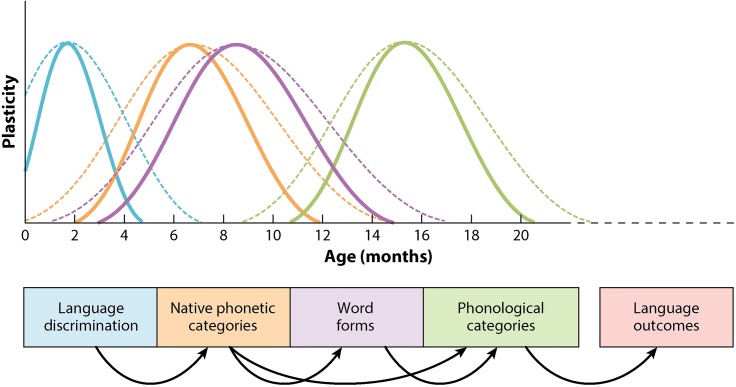
Sequential, overlapping critical periods in infant speech perception development. Solid lines represent typical onsets and offsets; broken lines indicate extensions of periods. Adapted from Werker and Hensch (2015). Republished with permission from Annual Reviews.

To get a fuller sense of the neurobiology of plasticity, and how it might relate to variability in language learning, it is instructive to connect critical-period research in the L1 context with studies in L2 acquisition and bilingualism. The essential neurobiological and experiential characteristics of early language learning are authoritatively laid out by Werker and Hensch (2015), who describe the cascading sequence of multiple, overlapping periods of plasticity that enable the development of phonetic perception in the native language, starting with discrimination of linguistic sounds in the first few months of infancy through the structuring of word forms and phonological categories as children approach 20 months of age; see **Figure 1**.

The chronologies of the onset, the duration, and the closure of each of the critical periods are not fixed, but are manipulated by biological and experiential factors. For example, the timing of the closure of critical periods depends on molecular brakes such as myelin and histone deacetylases, and onset timing can be delayed by sensory deprivation and maternal depression. Thus it is understood that variability and plasticity go hand in hand, as variability within and across overlapping periods of plasticity is a basic feature of the model.

Notably, at the level of the individual child the duration of critical periods in speech perception development can be varied through bilingual experience. As examples, Werker and Hensch cite studies showing that the duration of the critical period for perceptual narrowing – the process by which infants orient their emergent speech perception abilities around just those sounds that occur in their linguistic environment – is longer among simultaneous bilingual children than among monolingual children. The researchers point to several possibilities for this extension.

Relative to monolingual infants, among bilingual infants: native speech categories take longer to establish (Bosch and Sebastián-Gallés, 2003); sensitivities to speech sounds are maintained until an older age (Petitto et al., 2012); there is less input per language, with an asymmetric relative frequency of phones within and across the dual-language input (Bosch and Sebastián-Gallés, 2003); there is enhanced executive control and attentional function afforded by bilingualism (Kovács and Mehler, 2009); the neural circuitry supporting phonetic discrimination is less mature (Garcia-Sierra et al., 2011); the circuitry is equally mature but involves a different distribution of neural connections, with greater connectivity in prefrontal areas (Petitto et al., 2012).

At early developmental stages, the two languages of bilingual infants may resemble those of monolingual children. For example, Burns et al. (2007) found that, at 10–12 months, phonetic discrimination in both languages of English–French bilingual infants of resembled that of monolingual infants and lasted for several months thereafter. Once simultaneous and early bilinguals reach adulthood, however, their processing and production of speech differs from that of monolinguals in each language (see below). More to the point, among adult simultaneous and early bilinguals, variability in speech perception and production is widely attested, and the extent of differences among individuals is in general greater than that observed among native (monolingual) speakers; see Sebastián-Galles and Díaz (2012) for a review. This variability (which may reflect asymmetric exposure to or use of the two languages, or exposure to accented speech in one or both languages, along with motivation, context of learning, inter- individual neurobiological and neurocognitive differences over development, etc.; see further discussion below) is often demonstrated in behavioral studies through comparisons of early or simultaneous bilinguals with monolingual controls at local levels of analysis. For example, Mack (1989) looks at early English–French and French–English bilingual adults, all of whom were English dominant. For /ta/-/da/ discrimination and /i/-/I/ production, the bilingual group resembles English monolingual adult controls. In a separate analysis, however, for the percentage of /i/ vowels whose F2 fell at least 50 Hz between the vowel midpoint and offset, bilinguals differ significantly from monolinguals. Similarly, in Sundara et al.’s (2006) study of /d/-/t/ production, English–French simultaneous bilinguals resemble French monolinguals and English monolinguals for /d/ and /t/ in French and for /t/ in English, but diverge for English /d/.

## Plasticity, Variability and Critical Periods in L2 Acquisition

It is commonly believed that L2 attainment to nativelike levels among adults is impossible because they have passed a critical period for successful learning. Two general types of evidence are summoned to support this view. The first is the nature of the function that relates AoA to ultimate attainment. The second is evidence for comprehensive nativelike attainment across all aspects of knowledge, production, and processing of the L2.

### The AoA-L2 Attainment Function

**FIGURE 2:**
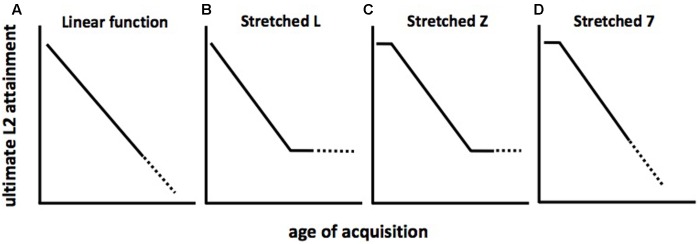
Schematic representations of age of acquisition (AoA) effects on L2 attainment. **(A)** linear decline of L2 attainment over all AoA; **(B)** initial decline of L2 attainment followed by leveling off over subsequent AoA; **(C)** L2 attainment plateau, followed by decline, followed by leveling off over subsequent AoA; **(D)** L2 attainment plateau followed by decline over subsequent AoA.

Theories of the geometry of the function that relates AoA to ultimate (asymptotic) L2 attainment are reviewed in Birdsong (2005) and Birdsong and Vanhove (2016). In brief, it is thought that departures from linearity in the function would suggest the effects of developmental events leading to qualitative changes in the neurocognitive mechanisms believed responsible for language learning (see Hakuta et al., 2003, for an overview). If instead the function is linear (**Figure 2A**), this would suggest other types of age-related effects. Some researchers have argued that declines in ultimate L2 attainment should level off after the end of maturation. That is, AoA effects on L2 attainment should be observed among early L2 learners, but AoA should no longer be predictive of L2 asymptote among post-adolescent learners, since maturation would presumably have ceased by this time. On this notion, the geometry of the function should resemble a “stretched L,” as seen in **Figure 2B**. On another view, L2 learning is successful up to a certain age (which may vary depending on what language features are being investigated), and learning ability (and, consequently, ultimate attainment) should decline thereafter. The corresponding shape of the function resembles a “stretched 7,” as shown in **Figure 2C**. A third geometry is that of a “stretched Z,” shown in **Figure 2D**, which combines the “L” and “7” features to include an early plateau, followed by a decline and floor.

To clarify, note that these are schematic representations only. Depending on methodological considerations (e.g., analysis over the AoA span vs. disaggregation by early and late AoA; choice of regression model, line fitting and smoothing methods, etc.) the observed shapes may have less angular features, and the slopes may be shallower. Also, the timing of the points along the AoA continuum where changes in slope are said to occur varies considerably from study to study. (For further discussion, see Birdsong, 2005; Meulman et al., 2015.)

The geometry and timing of AoA effects are crucial to the question of age-conditioned plasticity in L2 learning since, in order to be consistent with maturational effects, the inflection points on the function would need to match up with known maturational milestones. There are two main obstacles to establishing this isomorphism. One is that attained values on accent ratings and knowledge of morphosyntax map onto different functions. Divergences of L2 learners’ pronunciation from that of monolingual controls begin much earlier (even in the first year of life), relative to divergences from native controls for morphosyntax, which have been observed to begin anywhere from about AoA = 7 years to AoA = 27 years. While there is evidence for the intuitively appealing notion of “multiple critical periods” (Scovel, 1988; Granena and Long, 2013), it is a challenge to come up with a unified model that comprehensively aligns variable AoA effects with maturational milestones. Such an account would have to reckon with geometries that are known to vary depending on the pairings of the L1 and the L2, the particular linguistic structures being tested, and exposure, identificational, and motivational factors (e.g., Birdsong and Molis, 2001; see further discussion below). In these ways, considerations of plasticity and variability intersect.

Another challenge involves the analytical methods that are employed to generate the AoA- attainment function. Different statistical methods applied to the same data may result in different shapes of the function, thus introducing an additional dimension of variability in our conceptualization of plasticity. For example, in Johnson and Newport’s (1989) study of Chinese and Korean learners of L2 English, grammaticality judgment scores on a test of English morphosyntax declined linearly over AoA for learners with AoA ≤ 15 years (*r* = -0.87, *p* < 0.01). By contrast, for the later arrivals the scores were distributed more or less randomly (*r* = -0.16, ns), and the best-fitting line through the scatterplot of later-arrivals’ scores was roughly horizontal. (Note that this unsystematic dispersion was interpreted by Johnson and Newport as a flattening of the AoA-L2 attainment function; see below). In a subsequent reanalysis of the Johnson and Newport (1989) data, Elman et al. (1996) demonstrated that a single non-linear function accounts for about 63% of the variance over all participants’ scores, whereas separate linear regressions for younger and older arrivals account for only about 39% of the variance. Importantly, Elman et al. (1996) point out that the overall best-fitting curve produced by the non-linear model is visually a straight line, i.e., one with no apparent inflection or post-maturational leveling-off.

In a re-examination of the Johnson and Newport (1989) L2 grammaticality judgment data, Vanhove (2013) exposes problems with comparing the correlations for early- vs. late-arriving learners in order to infer maturational effects from different correlational slopes. For Johnson and Newport’s early arrivals, the slope of the correlation suggested a decline of scores over AoA, whereas for late arrivals the slope leveled off, with no subsequent AoA-related decline in performance. Together the two correlation slopes resembled a stretched “L” corresponding to one proposed version of a critical period for L2 acquisition. However, as noted above, the apparent “flattened” slope (as indicated by a roughly horizontal regression line) is the reflection of the high degree of variability in the performance of the late-arriving learners.

Vanhove (2013) attributes this essentially random dispersion of late learners’ scores to factors such as age-conditioned inter-individual differences in literacy, education, opportunities for L2 use, and motivation – that is, to factors unrelated to critical period constraints. Note as well that general performance levels are often predicted by such variables; see e.g., Birdsong (2014b), Hartshorne et al. (in press).

Vanhove (2013) also reanalyzes L2 grammaticality judgment data from DeKeyser et al. (2010), which involved two groups of Russian native speakers, one having emigrated to Israel and the other to the United States or to Canada. For both participant groups, DeKeyser et al. (2010) had found differences in correlation coefficients between AoA subgroups, and had interpreted the corresponding changes in slope as evidence of discontinuity consistent with critical period effects. In Vanhove’s reanalyses, linear and piecewise regressions each account for more than 60% of the variance for both the Israel and North American data. With a breakpoint set at AoA = 18 years, piecewise regressions revealed a linear decline for the Israel data, and only a slight departure from linearity in the North American data.

In contrast, using various regression models, some studies find a “stretched-7” geometry for the AoA-L2 attainment function. For example, Hartshorne et al. (in press) elicited grammaticality judgments for English from 669,498 respondents to an online survey, two-thirds of whom were learners of English from different native languages. The results reveal an L2 ultimate attainment plateau that extends from birth to AoA = 10–12 years, followed by an unbounded decline in judgment accuracy over the remaining AoA range. In a masked priming paradigm involving 94 Turkish–English bilinguals who had learned German at various ages, Veríssimo et al. (2017) observe nativelike priming for inflected German participle forms when the participants’ learning began before 5 years of age. After this plateau, facilitation declines with increasing AoA, with no leveling off. Another “stretched-7” geometry is noted by Birdsong and Molis (2001) in their replication study of Johnson and Newport (1989). For 61 native Spanish learners of L2 English, an ultimate attainment plateau terminates at a best-fitting inflection point at AoA = 27.5 years, and performance declines thereafter as AoA increases.

Meulman et al. (2015) illustrate the connectedness of analytical choices, the shape of the AoA-attainment function, and variability across structures under investigation. The researchers looked at ERP P600 signatures for the processing of violations of non-finite verbs and grammatical gender agreement in German by Slavic L1 speakers with advanced proficiency in German L2. AoA effects were not found for non-finite verb violations, which are similar in Slavic and German. However, among participants with AoA ≤ 20 years, gender violations elicited a P600, while among those with later AoA a posterior negativity was found in the same time window. Under Generalized Additive Modeling (GAM), and using both AoA and ERP time windows as continuous variables, linear AoA effects on EEG signals were observed across the AoA span, with no discontinuity in the function. Contrarily, ANOVA suggested a critical period prior to AoA = 17.

### Non-nativelike Attainment

As a second type of support for critical period effects in L2 acquisition, some researchers point to the lack of evidence for across-the-board nativelikeness in late L2 acquisition (e.g., Long, 1990; Hyltenstam and Abrahamsson, 2003; DeKeyser and Larson-Hall, 2005). The underlying logic is that language learning is biologically destined to be successful if begun in during a critical maturational epoch in early childhood, and that the failure of late learning to attain nativelike competence is the inevitable result of having passed a critical period of neural plasticity. Close comparisons of monolinguals and late L2 learners typically reveal differences across many dimensions of observation (e.g., Abrahamsson and Hyltenstam, 2009), and proponents of the Critical Period Hypothesis for L2 acquisition (CPH/L2A) posit that across-the-board monolingual-likeness is impossible. On this account, in order to falsify the CPH/L2A, one would have to identify at least one late L2 learner who is indistinguishable from a monolingual native across every imaginable measure of linguistic processing and knowledge (Long, 1990).

This argument is implausible, however, because the nature of bilingualism is such that the languages of an active bilingual are activated simultaneously (Dijkstra and Van Heuven, 2002; Schwartz and Kroll, 2006) and influence each other reciprocally (Grosjean, 1989; Cook, 1999, 2003; Flege et al., 2003). Given coactivation and bidirectional effects, neither the first nor the second language of bilinguals can be expected to resemble under scrutiny that of monolinguals in either language. Since “two monolinguals in one person” is an impossibility (Grosjean, 1989), it is unreasonable to hold up a standard of “across-the-board monolingual nativelikeness” in the L2 as a criterion for falsifying the CPH/L2A (Birdsong and Gertken, 2013).

Returning to the question of plasticity, it is important to keep in mind that the L1 is permeable in bilingualism; thus, considerations of plasticity apply to the L1 as well as the L2. The fact that the L2 influences the L1, not just the other way around, suggests that alleged adult L2 learning “deficits” (in the form of divergences from monolingual-likeness) should not be ascribed uniquely to a maturationally determined loss of plasticity. Moreover, the fact that it is not only late L2 learners who exhibit such differences, but early bilinguals and bilinguals-from-birth as well, is plausibly explained under a bilingualism effects account (e.g., MacLeod and Stoel-Gammon, 2005; Fowler et al., 2008; Ortega, 2009). (Note in this context that no researchers claim that bilingualism effects alone are responsible for all divergences from monolingual-likeness in bilingualism.)

Attested non-nativelikeness in both languages of an active bilingual has clear implications for theory. To the extent that an account of L2 acquisition predicts that L2 learners should not attain across-the-board nativelikeness if they have passed a biologically regulated critical period, it should also logically predict that the L1 of a bilingual, which is learned within that critical period, *should* exemplify monolingual-likeness across the board. However, this prediction is not borne out in the relevant research. By contrast, the evidence of bilingualism effects supports an account under which neither the L2 (irrespective of AoA) nor the L1 are completely monolingual-like. Note in this regard that the accuracy figures for bilinguals from birth are significantly lower than those of native monolingual controls in Hartshorne et al. (in press).

These observations connect straightforwardly to questions of age, plasticity, and variability. In their meta-analysis, Liu and Cao (2016) cite studies of L1 permeability in bilingualism, which reveal different patterns of neural activation in the L1 after vs. before acquisition of the L2. Introducing the AoA factor, several reviewed studies converge on the finding that early bilinguals, relative to late bilinguals – with both sampled populations having the same L1 – showed greater activation in the left fusiform gyrus than late bilinguals when processing the L1. This result suggests that the effects of the L2 on L1 processing in imaging studies may be more pronounced with earlier AoA of the L2, as the L2 ‘interferes’ more with the L1 to the extent that development of the two languages overlaps temporally. This relationship is attested as well in behavioral studies.

The basic notion that L2 ultimate attainment is conditioned by the age of initial immersion or significant exposure is examined by Qureshi (2016) in a meta-analysis of 26 studies of morphosyntactic knowledge. The materials reviewed largely substantiated the general idea of AoA effects (as opposed to maturational effects, which were not explicitly examined). At the same time, experiential and methodological factors were found to introduce considerable variability in outcomes. For example, in studies of classroom learning of a foreign language, there was no evidence of an “early advantage” (see also Huang, 2016), whereas the “early-is-better” rule of thumb was supported in studies of immersion learners.

### Nativelikeness

It is important to emphasize that, despite bilingualism effects, there are late L2 learners who resemble native monolinguals with respect to targeted aspects of the L2 (as opposed to bilinguals being indistinguishable from monolinguals in every measurable respect). Behavioral evidence ranges from acquisition of fine-grained phonetic features such as VOT to global pronunciation (Bongaerts, 1999; Flege et al., 2002; Birdsong, 2007; Moyer, 2014) and from surface morphology to abstract features of syntax (Birdsong, 1992; Birdsong and Molis, 2001; Donaldson, 2011; Destruel and Donaldson, 2017). In online tasks such as self-paced reading, late bilinguals show monolingual-like sensitivity to subtle and unique aspects of the L2 such as order of clitic pronouns (Rossi et al., 2017). In brain-based studies, high-proficient late L2 learners exhibit convergence with native participants (Green, 2003) in the processing of information structure (Reichle and Birdsong, 2014) and across a variety of syntactic and morpho- syntactic features: see Steinhauer (2014) for a review of the electrophysiological literature; see Abutalebi (2008) for a review of the functional neuroimaging literature.

The incidence of nativelikeness among late L2 learners can vary as a function of the particular structural characteristics that are investigated and as a function of the experimental procedures that are employed. For example, in a series of experiments that involved both ERP and eye-tracking methodologies, Foucart and Frenck-Mestre (2012) find that violations of noun-adjective gender agreement in French trigger nativelike P600 signatures among English-speaking late learners of L2 French when the adjectives follow the nouns, but elicit non-nativelike N400s when the adjectives are preposed. When the stimuli involve agreement violations in predicative structures (where the noun and the adjective are separated by a copula), natives and learners diverge in terms of ERP, but show similar patterns in eye tracking.

Birdsong and Gertken (2013) point out that the incidence of nativelikeness may depend on which native speakers the learners are being compared to. For example, Indefrey (2006), reviewing studies involving the processing of complex syntax, discerns that natives with high memory spans attend to structural features for correct interpretation in online tasks whereas natives with low memory spans rely on lexico-semantic information – as do many L2 speakers. Indefrey (2006, p. 68) argues that “non-structural sentence processing observed in L2 speakers is an option that is also used by native speakers when they have limited processing resources,” thus underscoring another type of variability inherent in assessing nativelikeness.

In some of these and related studies, the findings of nativelikeness have been interpreted as counter-evidence to critical-period predictions with respect to the attainment of nativelikeness in late L2 acquisition articulated. Recall, however, that proponents of the critical period hypothesis in the L2 context advance the criterion of across-the-board nativelikeness as necessary evidence for rejection of the hypothesis. From this perspective, among late (or early) bilinguals it is not enough to find “pockets” of nativelikeness with respect to grammatical knowledge and online processing, or brain activation patterns that resemble those of monolinguals, or individuals who diverge from controls only on VOT values for /d/ in word-final position, but in no other respect.

Under comprehensive, microscopic scrutiny, even among the most practiced hyper-polyglots (see section “Individual Differences in L2 Learning,” below), some scintilla of non-monolingual-likeness can be found among active bilinguals.

As stated above, however, the position regarding falsification of the hypothesis by impeccable nativelikeness does not take into account the natural effects of bilingualism, which make it impossible for *both early and late bilinguals* to be exactly like monolinguals *in either the L1 or the L2*. It was also noted that, by the logic of this position, for rejection of the nature-of-bilingualism account (and for support of the critical period account) one would need evidence of across-the-board monolingual-likeness in the first-learned language of late bilinguals, or in either language of simultaneous bilinguals (Birdsong and Vanhove, 2016).

## Sources of Variability in L2 Acquisition

### Two Illustrations

**FIGURE 3:**
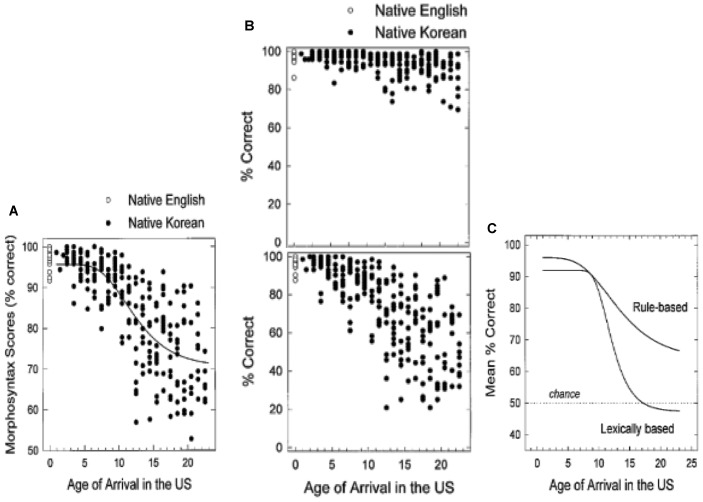
Results of a test of English morphosyntax, as a function of age of arrival in the United States. **(A)** Shows overall percent correct for Korean native speakers (filled circles) and native English controls (open circles); **(B)** breaks out test results by grammatical items (top) and ungrammatical items (bottom); **(C)** depicts different functions for ungrammatical rule-based items vs. ungrammatical lexically-based items. Adapted from Flege et al. (1999). Republished with permission from Elsevier.

Flege et al. (1999) provide an instructive illustration of factors that interact with AoA to produce distinct patterns of inter-subject variability within the function that relates AoA to L2 attainment. The researchers tested 240 Korean adults’ knowledge of L2 English morphosyntax with an adapted version of the Johnson and Newport (1989) materials. **Figure 3A** plots the Koreans’ overall performance (black circles) and that of native English controls (open circles). As seen in the plateau at ceiling, participants with early AoA (up to about 7 years of age) perform relatively homogeneously and within or close to the range of native controls.

With increasing AoA, the learners’ results become more dispersed. **Figure 3B** plots the L2 English performance on the same items, broken out by those that are grammatical (top image) and ungrammatical (bottom image). Both the top and bottom images reveal increased variability over AoA; however, the degree of variability depends on the grammatical status of the items analyzed, with the cone-shaped scatter of results more pronounced for responses to ungrammatical items than to grammatical items.

Another source of variability is the test items themselves, as shown in **Figure 3C**. For ungrammatical “rule-based” items that exemplify regular, generalizable features of English surface morphology (e.g., *–ed* past inflection on verbs; case marking on personal pronouns), the slope of the decline in performance over AoA is relatively shallow. By contrast, a steep decline over AoA is observed for ungrammatical “lexically-based” items that exemplify idiosyncratic features of English, such as prepositions preceding infinitival complements (^∗^let to watch vs. let watch) and noun complements (e.g., ^∗^hoping rain vs. hoping for rain). Note as well that the shape of the function for the ungrammatical lexical items roughly resembles the schematic “stretched-Z” geometry (see **Figure 2C**), while the function for the rule-based items is closer to linearity (see **Figure 2A**).

**FIGURE 4:**
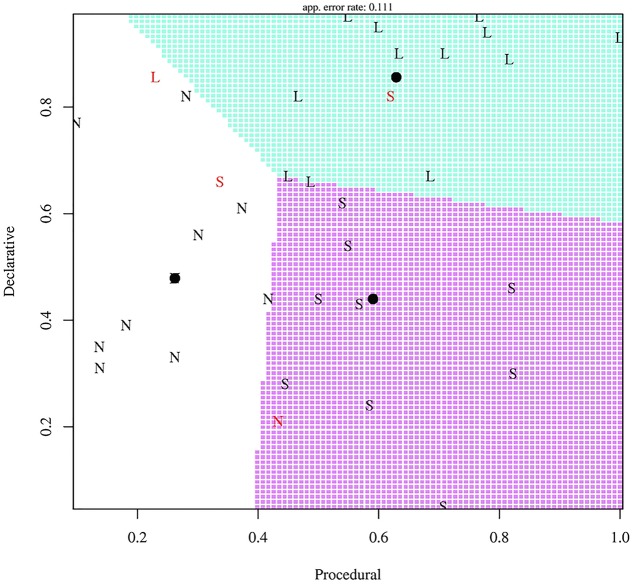
Performance on procedural and declarative memory tasks for Learners (L), Non-learners (N), and Simplifiers (S). Adapted from Ettlinger et al. (2014). Republished with permission from Cambridge University Press.

As a second illustration of sources of variability, Ettlinger et al. (2014) examine the possibility that L2 learner strategies and success vary according to domain-general cognitive skills. In an artificial language based on Shimakonde, a Bantu language of Mozambique, university student participants were trained on noun stems, plurals, diminutives, and diminutive plurals representing animals. For two types of diminutive plurals in the language, the diminutive and the plural morphemes are simply affixed on the singular stem. A third type of diminutive plurals is more complex, as the vowels in the stem and the plural affix require rephonologization. After exposure to word-picture pairs, participants were asked to produce diminutive plurals on novel words à la the *wug* test (Berko, 1958). Some learners (termed Simplifiers) tended to apply the simple pattern in instances of both complex and simple diminutive plurals; others (Learners) successfully learned both the complex and simple diminutive plurals; others (Non-learners) performed poorly overall. On a prior test of working memory, Learners, Simplifiers and Non-learners performed similarly. However, the groups varied on prior tests of procedural memory and declarative memory. Those participants who were Learners generally scored high on both procedural and declarative memory tests. Those with high procedural memory scores, but lower declarative memory scores, tended to be Simplifiers. Those with poor procedural memory, irrespective of declarative memory scores, were Non-learners. These results, summarized in **Figure 4**, suggest a connection between learner types and L2 learning performance: differences in domain-general cognitive capacities account for some inter-individual variation in L2 learning.

### Variability in L2 Attainment With Increasing AoA: Possible Sources

In some studies, as AoA increases, the outcome of learning of L2 morphosyntax appears to become more variable (see, e.g., Flege et al., 1999; Vanhove, 2013). Candidate sources for such wide dispersions can be inferred from an increase over age of the range of values that are associated with relevant experiential variables. For example, in a random participant sample, the range of lengths of residence in the L2 environment, along with the range of years and types of education will increase correspondingly with AoA. Along with such scaling effects on demographic variables, it is also possible that, with increasing AoA, motivation to attain accuracy in lexico-grammatical knowledge in L2 will become more heterogeneous across participants, particularly so as goals for L2 learning become more diverse.

Cognitive aging may also figure in the mix of candidate reasons for age-related variability in L2 attainment. For example, Buczylowska and Petermann (2016) summarize age-related differences in six executive function tests administered to 484 participants ranging in age from 18 to 99 years. Declines in mean scores over age were accompanied by increased age-dependent heterogeneity in scores. Connecting this finding to the cone-shaped dispersion of L2 morphosyntax scores over AoA is not a straightforward matter, however, as the heterogeneity observed by Buczylowska and Petermann (2016) is most notable in the later age ranges, whereas most individuals undertaking L2 do not begin so late in life. Further, the degree of dispersion varied greatly by task in this study. Similarly, Mella et al. (2016) show that results on tests of processing speed and working memory do not display the same inter-individual variability with increasing age. Relatedly, Hartshorne and Germine (2015) find that the peaks in cognitive skill are not synchronized over skill types, with some occurring earlier than others. The occurrence of multiple décalages in the timing of peaks (and subsequent declines) suggests that whatever scatter of performance there is over age may not be uniform over intelligence types.

A strong case can be made for both general effects and inter-individual effects of progressive cognitive decline, as well as for effects of dopamine declines (see above), progressive L1 entrenchment (Marchman, 1993; Elman et al., 1996; Flege, 1999; MacWhinney, 2005), and education (Bialystok and Hakuta, 1999; Birdsong, 2014b) on L2 attainment over AoA. At the same time, it is fair to say that further study is needed to establish a direct link between heterogeneity in cognitive function over age and AoA-related patterns of dispersion of results on tests of L2 attainment.

## Individual Differences in L2 Learning

It is axiomatic that people vary widely in the effectiveness and efficiency with which they learn an L2. Often the study of individual differences in L2 learning focuses on exceptionally successful learners. Although researchers do not all agree on terminological distinctions between the notions of ability, aptitude, talent, and giftedness in the context of L2 learning, the cognitive and conative attributes of high achievers in this domain are well understood; for a recent review, including the question of the mutability of aptitude with experience, see Singleton (2017).

Individuals who attain near-nativelikeness in multiple languages tend to be endowed with high working memory capacity, are highly motivated to learn, and strategically apply metalinguistic knowledge and analysis across their learned languages. In addition to these traits, “gifted multilinguals” score high on tests of intelligence and foreign-language learning aptitude, and are creative, persistent and self-aware (Biedroń and Pawlak, 2016). Polyglots – defined by Hyltenstam (2016) as those who reach high proficiency in six or more languages after puberty – and hyper- polyglots – for Erard (2012) those who proficiently speak, read, or write in at least 11 languages – share the same traits as gifted multilinguals, while also possessing extraordinary verbal memory. They apply their superior analytic skills to recognize patterns in phonology and morphosyntax, and with remarkable executive control are able to switch between languages with little interference. The linguistic savant Christopher (Smith et al., 2011), who has learned more than 20 languages, exhibits autistic traits and accordingly differs from polyglots and hyperpolyglots in terms of cognitive neurostructure. Pring (2007) notes that autistic savants also differ behaviorally from non-autistic experts by their obsession with memorization and practice, which appears to be more about the pleasure of obsessiveness than about achievement. According to Pring, it is typical of high achievers, but not of savants, to strategically set goals and to use feedback when learning.

Biedroń and Birdsong (in press) point out that, to the extent that complete monolingual nativelikeness is taken to be criterial, extraordinary polyglots do not constitute so-called “exceptions to the critical period hypothesis” for L2 acquisition. As suggested above, it is more apposite to point out that there are no exceptions to the effects of bilingualism, even among the most talented learners of languages. As Biedroń and Birdsong observe, “the special significance of the impossibility of multiple monolingual-likenesses resides in the fact that, no matter how gifted a multilingual is, s/he can’t suppress in an absolute sense the other language(s).”

Turning to less exceptional cases, Della Rosa et al. (2013) proffer a view of individual talent in multilingualism that relates language-learning-induced plasticity in the left inferior parietal (LIPL) region of the brain to enhancement of domain-general attentional processes. Their longitudinal study of children living in the South Tyrol region of Italy, where German, Italian, Ladin and English are routinely used, showed specific multilingualism-induced gray matter volume increases in the LIPL. The researchers suggest that such structural adaptations result from the necessity to apply general memory and attentional functions to the processing of more than one language.

A neurogenetic approach to individual differences in L2 learning is advanced by Wong et al. (2012), who specify the mediating roles of genetically encoded dopaminergic (DA) reception and transmission that underlie the acquisition of procedural aspects of grammar. Procedural learning is associated with concatenation of constituents in syntax and with abstract relations between phonology and morphology, and is localized in the prefrontal cortex and basal ganglia. Given what is known about idiosyncratic variability in DA-related gene function, expression and biochemistry, “it is not surprising that individuals with different genetic profiles may have different learning capabilities” (Wong et al., 2012, p. 1093), with more variation expected in adult L2 acquisition than in L1 acquisition. These differences extend to inhibitory function and executive control, which in L2 processing enable suppression of competing information such as knowledge and intrusion of the L1 (Lee, 2004). Under the DA account, a mediating role of AoA can be postulated as well, as dopamine receptor and binding declines over age are well documented (e.g., Volkow et al., 1998; Prull et al., 1999; Bäckman and Farde, 2005).

Taking this approach to variation a step farther, Wong et al. (2017) examine behavioral, neural, and genetic predictors of learning at the level of the individual, and discuss the applications of personalized learning in the L2 context. Drawing parallels with personalized medicine in the pharmacological field, the authors suggest that understanding individual differences will lead to customization and optimization of language instruction. For other studies of individual differences in cognitive abilities (in particular, differences in procedural, declarative and working memory), and how these play out in second language acquisition, see Morgan-Short et al. (2014) and Faretta-Stutenberg and Morgan-Short (2018).

A dual-systems learning model developed by Chandrasekaran et al. (2014) looks at the use of reflexive vs. reflective learning systems in speech category learning in a training paradigm. The reflective system explicitly develops and tests categorization rules; in contrast, the nature of the reflexive system is procedural and implicit. In experiments involving novel linguistic tone category learning, adult participants initially display a bias toward using the reflective system, which turns out to be ill-adapted to the task. Those individuals who succeed in tone learning are able to shift to the reflexive system, using cortico-striatal connections whose plasticity is regulated by DA reinforcement signals. Relative to younger participants, older adults appear to be less likely to be able to shift from reflective learning to reflexive learning.

Birdsong (2012) examines native-language literacy and education as sources of variability across participants in L2 attainment studies. These factors may interact with task type (e.g., grammaticality judgments vs. truth-value tasks; elicited speech vs. read-alouds), measure (e.g., behavioral vs. brain-based measures; speed vs. accuracy) and linguistic domain (e.g., quantifier scope, garden-path structures). Birdsong (2012) also notes that both native speakers and L2 learners exhibit grammatical idiosyncrasies and other types of variability in representations of linguistic structure (Dabrowska, 2012); therefore variability *per se* (whatever the type or source) is not necessarily evidence of learning deficiencies.

According to Birdsong (1994), the ability to make judgments about linguistic form differs across individuals, who vary in the way they construct language-relevant categories such as “well- formed sentence” and “plausible interpretation.” Individual learners may also differ in assessments of the typological relatedness of their L1 to their L2, which modulates their decisions about the likelihood that features of their L1 will resemble those of their L2. Birdsong (2009) characterizes individual differences in learners’ ability to notice subtle linguistic features of the L2 within the general framework of signal-detection theory.

For an overview of individual variation in L2 processing (as opposed to attainment), see Van Hell and Abdollahi (2017).

## Dominance, Plasticity, Variability and Age

A feature of bilingualism that conspicuously connects age, plasticity and variability is linguistic dominance. Regarding plasticity and age, it is not always the case that language learned in infancy is the dominant language of a bilingual: the neural mechanisms involved are sufficiently plastic that the L2 can “leapfrog” the L1 in terms of proficiency and processing ease. Among international adoptees and heritage speakers, dominance shifts involve attrition of the L1, a representational and functional *loss* which likewise reflects neural plasticity (see below). As concerns variability, inter-individual differences in dominance relationships are natural consequences of idiosyncratic experiences with, skills in, and use of the two languages. No two bilinguals are identical in terms of dominance.

Linguistic dominance in bilingualism is understood in terms of dimensions – relative performance in a language skill such as speech rate, picture naming or grammatical accuracy – and in terms of domains – typically, the comparative frequency of use of each language at work, with family members, or at school. Dominance is not uniquely equatable with relative proficiency (as defined in terms of grammatical and lexical accuracy, speech fluency, etc.), since there are other dimension-based measures of dominance besides proficiency (e.g., object naming speed, lexical diversity, reading speed). Relatedly (and to underscore the dimension/domain distinction in dominance measures), a bilingual parent who is L1-dominant in terms of lexical knowledge and fluency of speech may by choice use the L2 in all interactions with offspring who are being raised in that language, thus demonstrating domain-based L2 dominance in this particular context of use. For further discussion and evidence relating to the independence of dominance and proficiency, see Luk and Bialystok (2013), Montrul (2016a), and Schmeißer et al. (2016); also discussion of balanced bilinguals below.

As with many other features of bilingualism, linguistic dominance is not inherently categorical. That is, individual bilinguals are not simply “L1-dominant” or “L2-dominant,” they are dominant in one or the other language to varying degrees. Accordingly, in order to faithfully capture the construct, dominance, like AoA, is properly operationalized and analyzed as a continuous subject factor. As with any other continuous variable, participant assignment to dominance categories may mask intra-group variability and result in loss of statistical power (e.g., Altman, 1998). Some instruments for assessing dominance take into account both domains and dimensions of dominance. Birdsong (2016) reviews methods of calculating dominance indices, along with problems of incommensurability in comparing individual bilinguals who may have the same composite dominance indices, but who vary with respect to the underlying dimensions and domains measured by the instrument.

### Balanced Bilingualism

**FIGURE 5:**
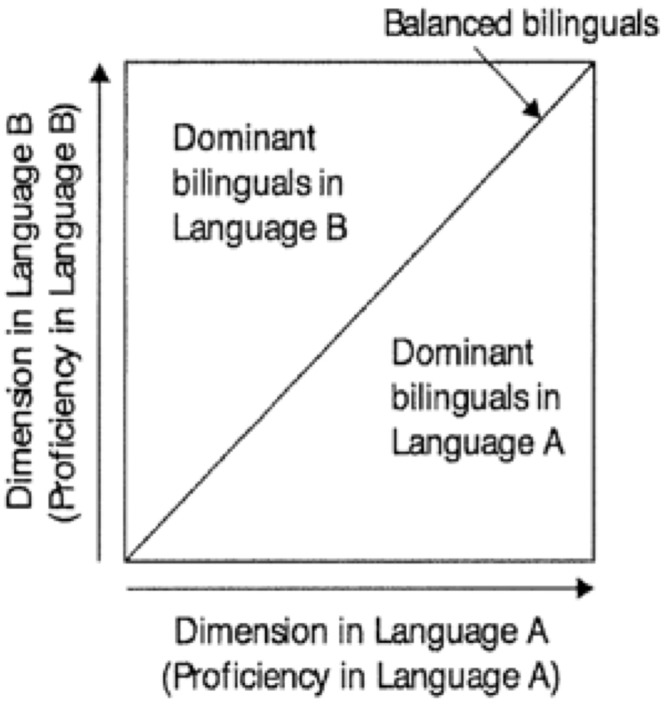
Representation of balanced bilingualism, showing that “balanced” only implies dominance in neither language, not high proficiency in both languages. Adapted from Goto Butler and Hakuta (2004). Republished with permission from John Wiley and Sons.

So-called “balanced bilinguals” are dominant in neither language. The term is sometimes used or assumed to denote very high or (near-)nativelike proficiency in both languages. However, degree of proficiency is independent from degree of dominance. An individual who is at an equally low proficiency level in two languages, and an individual who is highly and equally proficient in two languages, are both by definition balanced bilinguals. As depicted by Goto Butler and Hakuta (2004), **Figure 5** shows that balanced bilinguals fall at any point along the diagonal line of increasing proficiency. Bilinguals who are not balanced (that is, who are dominant in either Language A or Language B) are situated to one side or the other of the diagonal.

The related idea of “perfect bilingualism,” if understood as monolingual-likeness in two languages, is misguided since, as noted earlier, neither the L1 nor the L2 of bilinguals is identical to the corresponding languages of monolinguals in all measurable respects. Becoming “more bilingual” is, however, sometimes thought to suggest getting closer to “perfect bilingualism.” At the same time “more bilingual” has been taken to mean that a given bilingual is highly proficient in the two languages, or to mean approaching balanced proficiency.

### Dominance Shifts and Age

The direction and degree of dominance in the two languages are dynamic over the lifetime of an individual bilingual. Depending on changing circumstances such as immigration, educational and occupational opportunities, and psycho-social identification, the L2 may “replace” the L1 as the dominant language. In some cases, and for similar reasons, the L1 may return to dominance, and still further shifts are possible. Grosjean (2010) details multiple dominance shifts over 60 years of his life. For a review of research and theory on the relationship between dominance and age, see Birdsong (2014a).

Conceptually as well as in practice, the developmental dynamics of dominance relationships may reflect both L1 loss and L2 gains. For example, among some immigrants and adoptees, there may be little or no ongoing use of the L1; as the L1 withers (in terms of domains or dimensions), the L2 perforce becomes the dominant language. On a developmental scenario, a sequential bilingual whose L1 is not fully developed may use and maintain the L1, but as a matter of relative gains in linguistic knowledge and proficiency over time, the L2 eventually outstrips the L1.

Losses in the L1 and gains in the L2, with consequent reflexes in the dominance relationship between the two languages, have been theorized together in terms of maturational constraints on plasticity. Bylund et al. (2012) propose that the same maturational mechanisms synchronously constrain both the ability to lose a language and the ability to gain a new language. Bylund et al. (2012) state that the potential for L1 attrition and the potential for L2 attainment are highest during the first 10 years of life. After this period the potential for both L1 attrition and L2 attainment declines, with the relevant geometry of both resembling a stretched-7. Pallier (2007) advances a different view, whereby the AoA-ultimate L2 attainment function exhibits a linear decline, starting essentially at birth; by contrast, the likelihood and degree of L1 attrition start to drop off only after age 10. For Pallier (2007) and Bylund et al. (2012) alike, plasticity for both L1 attrition and L2 attainment are age conditioned; however, for Pallier the age effects for L2 attainment do not correspond to maturational effects in the AoA-attainment function, as there is no departure from linearity along the function that would suggest a qualitative change in learning ability.

Thus, with respect to plasticity in dominance relationships in the first decade of life, there are two distinct possibilities. One possibility is that L1 loss, the likelihood of which is highest for several early years, is a greater contributor to dominance shifts than L2 gains, which start to become less likely very early in life, with progressively less influence on shifts from L1 to L2 dominance. Another possibility is that L2 gains and L1 losses conspire simultaneously to enable L1-to-L2 shifts of dominance. The latter possibility relates L1 loss and L2 gain under a unified view of plasticity in early childhood development: “the ease with which an L2 is acquired and the L1 undergoes attrition can be said to be manifestations of a generally heightened responsiveness to language exposure, which works both in acquisitional and attritional directions” (Bylund et al., 2012, p. 237). For a recent empirical study and review of age effects on L1 attrition, see Ahn et al. (2017).

Note that age conditions not only the probability of L1 loss, but also the speed at which attrition occurs (Köpke and Schmid, 2004). As L1 loss slows, the point at which a complete shift to L2 dominance can be expected is delayed. Similarly, depth of attrition (the degree to which a domain or dimension is diminished) and breadth of attrition (the number of dimensions and domains diminished) should decrease with the age at which the loss begins. Thus, indirectly through L1 loss, age contributes to variability in L1–L2 dominance relationships (see also Montrul, 2016b).

### Examples of Prediction and Variation in Dominance

**FIGURE 6:**
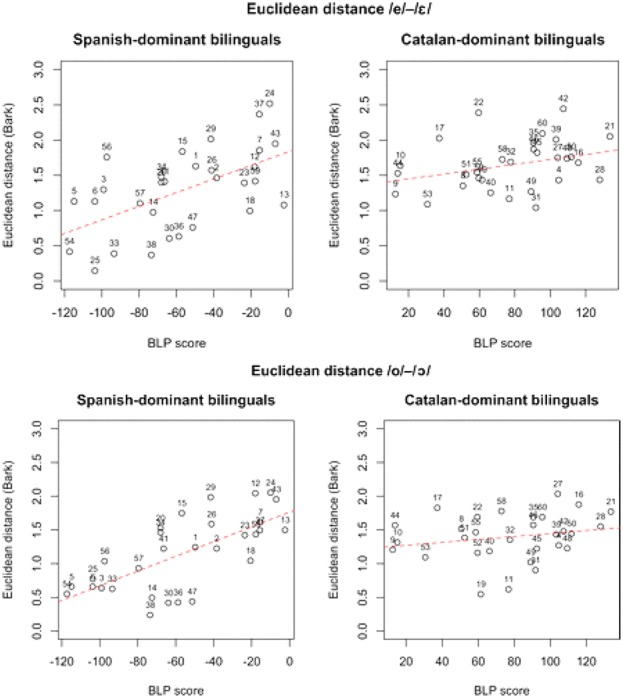
For Spanish-dominant and Catalan-dominant Majorcan bilinguals, Euclidean distances between same-speaker tokens of /e/-/ε/ and /o/-/ɔ/, plotted as a function of BLP scores, which range from –120 (strongest Spanish dominance) to +130 (strongest Catalan dominance). Adapted from Amengual (2014). Republished with permission from Sage Publishing.

Dominance has been shown to be a predictive factor in studies of bilingualism. As an example, Amengual (2014) looks at the elicited production of mid vowels among Spanish–Catalan bilinguals in Majorca. Catalan, but not Spanish, makes a phonemic distinction between the tense-lax mid /e/ - /ε/ and /o/ - /ɔ/. Relative Catalan vs. Spanish dominance was assessed with the Bilingual Language Profile (BLP; Birdsong et al., 2012). For the 30 Catalan-dominant bilinguals, degree of dominance was not predictive of the Euclidian distance between /e/ and /ε/ nor between /o/ and /ɔ/. However, among the 30 Spanish dominants, those whose BLP scores approached balanced bilingualism (i.e., whose scores were least Spanish dominant) produced the vowels with Euclidean distances resembling those produced by the Catalan dominants. Specifically, the BLP scores of Spanish dominants were predictive of more Catalan-like Euclidean distance between /e/ and /ε/ and between /o/ and /ɔ/ (see **Figure 6**).

**FIGURE 7:**
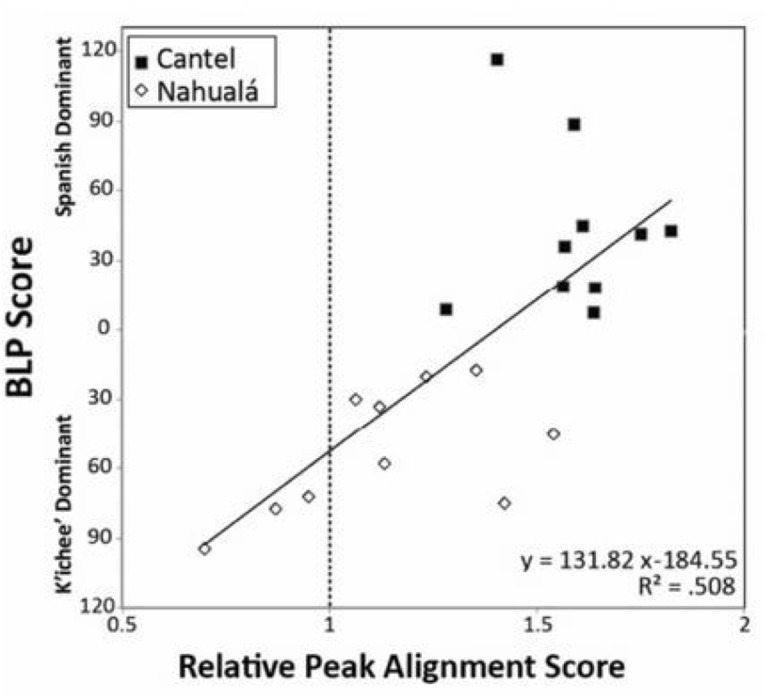
Correlation of BLP scores with relative peak alignment scores for Spanish–K’ichee’ bilinguals from Cantel and Nahualá. Zero-upward BLP scores = increasing Spanish dominance. Zero-downward BLP scores = increasing K’ichee’ dominance. Relative peak alignment scores = duration from syllable onset to peak pitch divided by total duration of syllable; values are individual speaker averages. Adapted from Baird (2015). Republished with permission from Cambridge University Press.

A study of bilingual speakers in Guatemala by Baird (2015) illustrates how the dominance factor accounts for inter-individual variation in bilingualism. Baird examines the pronunciation of Spanish tonic syllables by Spanish–K’ichee’ bilinguals from two Guatemalan communities, Cantel and Nahualá. In most varieties of Spanish, the peak of F0 rise occurs after the tonic syllable. In contact and bilingualism contexts, Spanish varieties display an F0 that is closer to (sometimes before) the tonic syllable. In a task involving reading Spanish phrases, 10 Spanish monolinguals, 10 bilinguals from Cantel, and 7 of the 10 bilinguals from Nahualá produced late (post-stress) F0 peaks. At the same time, for speakers from both communities, the degree of Spanish vs. K’ichee’ dominance, as assessed by the BLP, was predictive of the direction and distance of F0 peak placement; see **Figure 7** (Baird, 2015).

A critical take-away from Baird (2015) is that the nature of inter-individual variation is obscured in a simple analysis by binary factors, in this instance place of residence and pre- vs. post-stress F0 peaks. More revealing can be examinations of variation along continuous dimensions, in this case distance of peaks from the tonic syllable and degree of Spanish vs. K’ichée dominance. By such an analysis, individual variability along a continuum of peak F0 placement is predicted by degree of dominance, independently of residence.

Researchers have considered the possibility that dominance in the L2 may be associated with monolingual nativelikeness in pronunciation in that language. In a delayed sentence-repetition task for English sentences, Flege et al. (2002) found that Italian–English bilinguals who were L2-English dominant were judged not to have foreign accents, and suggested that L1-interference effects might be absent among L2-dominant bilinguals. A series of follow-up studies by Antoniou and colleagues look more closely at interference effects, in this case with respect to VOT among L2 dominants. In a sample of Greek–English sequential bilinguals who were L2 English-dominant, Antoniou et al. (2010) find that stop voicing among the L2-dominants mostly match that of natives in both languages, with exceptions for some L2 English medial stops reflecting a measure of L1 Greek interference. For the same bilinguals, Antoniou et al. (2011) examine VOT in code-switching between English and Greek. In contrast to the unilingual mode (one language activated) results of Antoniou et al. (2010), English stops in bilingual mode (both languages activated) are produced with more Greek-like values, whereas Greek stops do not display English-like VOT. That is, the L1 appears to influence pronunciation in the dominant L2, but not the other way around. Perception experiments with a larger sample of Greek–English bilinguals (Antoniou et al., 2012, p. 592) reveal a still more complex pattern of dominance relationships, one that depends on whether the task is categorization or discrimination of voicing: “The results suggest that a bilingual is a single (dominant-language) listener with respect to discrimination, but behaves more like a monolingual of the activated language with respect to discrimination judgments.” Taken together, the findings of Flege and colleagues and those of Antoniou and colleagues suggest a high degree of variability in terms of monolingual-like performance among L2-dominant bilinguals. Results may vary according to production vs. perception, language mode (unilingual vs. bilingual), task (discrimination vs. categorization), and level of analysis (global pronunciation vs. VOT).

Another illustration of the role of dominance in bilingualism relates to the question of executive control. A considerable body of research (e.g., Bialystok et al., 2012) suggests that enhanced executive control is conferred by bilingualism. At the same time, since bilingualism is not a unitary phenomenon and thus not a categorical variable (Luk and Bialystok, 2013); Yow and Li (2015) examine degree of dominance as a predictor of cognitive control within bilingual populations. Among 72 English–Mandarin young adult bilinguals, the researchers find a positive effect for balanced use and balanced proficiency with respect to interference in Stroop task performance and mixing cost in a number-letter (mental-set shifting) task. In addition, early AoA of the second language is associated with less interference on the Stroop task.

As a related and final example, recent work by Onnis et al. (2018) looks at dominance as a predictor of statistical learning among adult bilinguals in a miniature grammar paradigm. (Statistical language learning involves tracking the frequencies of, or the transitional probabilities between, grammatical elements, which results in implicit knowledge of structural regularities.) In this study, success in statistical learning of artificial grammars is predicted by the degree to which participants approach or depart from balanced bilingualism, as measured by BLP scores: balanced bilinguals perform better than those who are increasingly dominant in their first language. Thus, degree of bilingual dominance in adulthood is associated with differential ability to learn a novel language. Onnis et al. (2018, p. 432) summarize their findings: “By capitalizing on the bilingual variability we found in the [BLP] questionnaire rather than ignoring it, we unearthed important individual differences that point to the first documented modulating role of [degree of dominance in] bilingualism in adult statistical learning.”

## Conclusion

In this review we have seen how variation in L2 acquisition and bilingualism is conditioned by age, which itself conditions plasticity. We also know that age similarly conditions individual factors such as language experience, L1 attrition and linguistic dominance, which are themselves predictive of variation.

Age-related effects (of which neurobiological maturation within a critical period is one possible source) cannot account for all varieties of non-nativelike outcomes in L2 acquisition, since departures from monolingual-likeness are found not just in post-childhood learning but among from-birth simultaneous bilinguals as well. By contrast, bilingualism effects can account for observed non-monolingual-likeness in both the L1 and the L2, whatever the age of learning. At the same time, the degree of L1 activation, L1 entrenchment, L1 attrition and relative L1–L2 dominance – all of which are affected by AoA – modulate attainment levels across L2 learners.

The application of different statistical models and methods can result in different shapes of the function that relates AoA to L2 outcomes; such artifacts add another dimension of variability to the picture of L2 acquisition. We have also considered possible sources of variability in L2 attainment with increasing AoA. These sources range from experiential (education, length of residence), to representational (L1 entrenchment) and to cognitive decline with underlying neurologic causes such as dopamine levels that mediate domain-general learning and processing. The role of cognitive decline in AoA-related variability in L2 learning outcomes is of particular interest for future investigation.

This review has brought these concerns into focus with illustrations from two areas of active research, individual differences and bilingual dominance. With respect to individual differences in L2 learning, we have highlighted the roles of neurogenetic makeup, higher-order cognitive factors, language experience, age-conditioned learning styles and motivation. We have seen that the gradient phenomenon of dominance in bilingualism is dynamic over the lifespan, is conditioned by experience as well as by neural plasticity, and is predictive of phonetic variation, cognitive control, and statistical learning in artificial language paradigms.

In his classic position paper Bley-Vroman (1990, p. 13) problematizes adult L2 learning in terms of explaining “the quite high level of competence that is clearly possible in some cases, while also permitting the wide range of variation that is observed.” By demonstrating the connectedness of non-uniform outcomes with age and plasticity, the research reviewed here has shown that such variation is neither unexplainable nor unexpected. From this understanding emerges heuristic guidance for further explorations of the richness of L2 acquisition and bilingualism.

## Author Contributions

The author confirms being the sole contributor of this work and approved it for publication.

## Conflict of Interest Statement

The author declares that the research was conducted in the absence of any commercial or financial relationships that could be construed as a potential conflict of interest.

## References

[B1] Abrahamsson N. Hyltenstam K. (2009). Age of onset and nativelikeness in a second language: listener perception versus linguistic scrutiny. *Lang. Learn.* 59 249–306. 10.1111/j.1467-9922.2009.00507.x

[B2] Abutalebi J. (2008). Neural aspects of second language representation and language control. *Acta Psychol.* 128 466–478. 10.1016/j.actpsy.2008.03.014 18479667

[B3] Ahn S. Chang C. B. DeKeyser R. Lee-Ellis S. (2017). Age effects in first language attrition: speech perception by Korean-English bilinguals. *Lang. Learn.* 67 694–733. 10.1111/lang.12252

[B4] Altman D. G. (1998). “Categorizing continuous variables,” in *Encyclopedia of Biostatistics* eds Armitage P. Colton T. (New York, NY: Wiley), 563–567.

[B5] Amengual M. (2014). The perception and production of language-specific mid-vowel contrasts: shifting the focus to the bilingual individual in early language input conditions. *Int. J. Biling.* 20 133–152. 10.1177/1367006914544988

[B6] Antoniou M. Best C. T. Tyler M. D. Kroos C. (2010). Language context elicits native-like stop voicing in early bilinguals’ productions in both L1 and L2. *J. Phon.* 38 640–653. 10.1016/j.wocn.2010.09.005 21743759 PMC3131106

[B7] Antoniou M. Best C. T. Tyler M. D. Kroos C. (2011). Inter-language interference in VOT production by L2-dominant bilinguals: asymmetries in phonetic code-switching. *J. Phon.* 39 558–570. 10.1016/j.wocn.2011.03.001 22787285 PMC3391600

[B8] Antoniou M. Tyler M. D. Best C. T. (2012). Two ways to listen: do L2-dominant bilinguals perceive stop voicing according to language mode? *J. Phon.* 40 582–594. 10.1016/j.wocn.2012.05.005 22844163 PMC3403831

[B9] Bäckman L. Farde L. (2005). “The role of dopamine systems in cognitive aging,” in *Cognitive Neuroscience of Aging: Linking Cognitive and Cerebral Aging* eds Cabeza R. Nyberg L. Park D. (New York, NY: Oxford University Press), 58–84.

[B10] Baird B. O. (2015). “Pre-nuclear peak alignment in the Spanish of Spanish-K’ichee’ (Mayan) bilinguals,” in *Proceedings of the 6th Conference on Laboratory Approaches to Romance Phonology* eds Willis E. W. Martín Burtagueño P. Herrera Zendejas E. (Somerville, MA: Cascadilla Proceedings Project), 163–174.

[B11] Berko J. (1958). The child’s learning of English morphology. *Word* 14 150–177. 10.1080/00437956.1958.11659661

[B12] Bialystok E. Craik F. I. Luk G. (2012). Bilingualism: consequences for mind and brain. *Trends Cogn. Sci.* 16 240–250. 10.1016/j.tics.2012.03.001 22464592 PMC3322418

[B13] Bialystok E. Hakuta K. (1999). “Confounded age: linguistic and cognitive factors in age differences for second language acquisition,” in *Second Language Acquisition and the Critical Period Hypothesis* ed. Birdsong D. (Mahwah, NJ: Lawrence Erlbaum Associates), 161–181.

[B14] Biedroń A. Birdsong D. (in press). “Highly proficient and gifted bilinguals,” in *Handbook of Bilingualism* eds Ortega L. De Houwer A. (Cambridge: Cambridge University Press).

[B15] Biedroń A. Pawlak M. (2016). New conceptualizations of linguistic giftedness. *Lang. Teach.* 49 151–185. 10.1017/S0261444815000439

[B16] Birdsong D. (1992). Ultimate attainment in second language acquisition. *Language* 68 706–755. 10.1353/lan.1992.0035

[B17] Birdsong D. (1994). Decision making in second language acquisition. *Stud. Second Lang. Acquis.* 16 169–182. 10.1017/S0272263100012869

[B18] Birdsong D. (2005). “Interpreting age effects in second language acquisition,” in *Handbook of Bilingualism: Psycholinguistic Approaches* eds Kroll J. F. de Groot A. M. B. (New York, NY: Oxford University Press), 109–127.

[B19] Birdsong D. (2006). Age and second language acquisition and processing: a selective overview. *Lang. Learn.* 56 9–49. 10.1111/j.1467-9933.2006.00353.x 21489393

[B20] Birdsong D. (2007). “Nativelike pronunciation among late learners of French as a second language,” in *Language Experience in Second Language Speech Learning: In Honor of James Emil Flege* eds Bohn O.-S. Munro M. J. (Amsterdam: John Benjamins), 99–116.

[B21] Birdsong D. (2009). Uninterpretable features: psychology and plasticity in second language learnability. *Second Lang. Res.* 25 235–243. 10.1177/0267658308100285

[B22] Birdsong D. (2012). Three perspectives on non-uniform linguistic attainment. *Linguist. Approaches Biling.* 2 255–259. 10.1075/lab.2.3.02bir

[B23] Birdsong D. (2014a). Dominance and age in bilingualism. *Appl. Linguist.* 35 374–392. 10.1093/applin/amu031

[B24] Birdsong D. (2014b). “The critical period hypothesis for second language acquisition: tailoring the coat of many colors,” in *Essential Topics in Applied Linguistics and Multilingualism: Studies in Honor of David Singleton* eds Pawlak M. Aronin L. (New York, NY: Springer), 43–50.

[B25] Birdsong D. (2016). “Dominance in bilingualism: foundations of measurement, with insights from the study of handedness,” in *Language Dominance in Bilinguals: Issues of Measurement and Operationalization* eds Silva-Corvalán C. Treffers-Daller J. (Cambridge: Cambridge University Press), 85–105. 10.1017/CBO9781107375345.005

[B26] Birdsong D. (2017). “Critical periods,” in *Oxford Bibliographies in Linguistics* ed. Aronoff M. (New York, NY: Oxford University Press).

[B27] Birdsong D. Gertken L. M. Amengual M. (2012). *Bilingual Language Profile: An Easy-to-Use Instrument to Assess Bilingualism.* Austin, TX: COERLL, University of Texas at Austin. Available at: https://sites.la.utexas.edu/bilingual/

[B28] Birdsong D. Gertken L. M. (2013). In faint praise of folly: a critical review of native/non-native comparisons, with examples from native and bilingual processing of French complex syntax. *Lang. Interact. Acquis.* 4 107–133. 10.1075/lia.4.2.01bir

[B29] Birdsong D. Molis M. (2001). On the evidence for maturational constraints in second-language acquisition. *J. Mem. Lang.* 44 235–249. 10.1006/jmla.2000.2750

[B30] Birdsong D. Vanhove J. (2016). “Age of second-language acquisition: critical periods and social concerns,” in *Bilingualism Across the Lifespan: Factors Moderating Language Proficiency* eds Nicoladis E. Montanari S. (Washington, DC: American Psychological Association), 162–181.

[B31] Bley-Vroman R. (1990). The logical problem of foreign language learning. *Linguist. Anal.* 20 3–50. 10.1017/CBO9781139524544.005

[B32] Bongaerts T. (1999). “Ultimate attainment in L2 pronunciation: the case of very advanced late learners,” in *Second Language Acquisition and the Critical Period Hypothesis* ed. Birdsong D. (Mahwah, NJ: Lawrence Erlbaum Associates), 133–159.

[B33] Bosch L. Sebastián-Gallés N. (2003). Simultaneous bilingualism and perception of a language-specific vowel contrast in the first year of life. *Lang. Speech* 46 217–243. 10.1177/00238309030460020801 14748445

[B34] Buczylowska D. Petermann F. (2016). Age-related differences and heterogeneity in executive functions: analysis of NAB executive functions module scores. *Arch. Clin. Neuropsychol.* 31 254–282. 10.1093/arclin/acw005 26953227

[B35] Burns T. C. Yoshida K. A. Hill K. M. Werker J. F. (2007). The development of phonetic representation in bilingual and monolingual infants. *Appl. Psycholinguist.* 28 455–474. 10.1017/S0142716407070257

[B36] Bylund E. Abrahamsson N. Hyltenstam K. (2012). Does first language maintenance hamper nativelikeness in a second language? *Stud. Second Lang. Acquis.* 34 215–241. 10.1017/S0272263112000034

[B37] Chandrasekaran B. Koslov S. R. Maddox W. T. (2014). Toward a dual-learning systems model of speech category learning. *Front. Psychol.* 5:825. 10.3389/fpsyg.2014.00825 25132827 PMC4116788

[B38] Cook V. (1999). Going beyond the native speaker in language teaching. *TESOL Q.* 33 185–209. 10.2307/3587717

[B39] Cook V. (ed.) (2003). *Effects of the Second Language on the First.* Clevedon: Multilingual Matters.

[B40] Dabrowska E. (2012). Different speakers, different grammars: individual differences in native language attainment. *Linguist. Approaches Biling.* 2 219–253. 10.1075/lab.2.301dab

[B41] DeKeyser R. Alfi-Shabtay I. Ravid D. (2010). Cross-linguistic evidence for the nature of age effects in second language acquisition. *Appl. Psycholinguist.* 31 413–438. 10.1017/S0142716410000056

[B42] DeKeyser R. Larson-Hall J. (2005). “What does the critical period really mean?,” in *Handbook of Bilingualism: Psycholinguistic Approaches* eds Kroll J. F. Groot A. M. B. de (New York, NY: Oxford University Press), 88–108.

[B43] Della Rosa P. A. Videsott G. Borsa V. M. Canini M. Weekes B. S. Franceschini R. (2013). A neural interactive location for multilingual talent. *Cortex* 49 605–608. 10.1016/j.cortex.2012.12.001 23294573

[B44] Destruel E. Donaldson B. (2017). Second language acquisition of pragmatic inferences: evidence from the French *c’est*-cleft. *Appl. Psycholinguist.* 38 703–732. 10.1017/S014271616000400

[B45] Dijkstra A. Van Heuven W. J. B. (2002). The architecture of the bilingual word recognition system: from identification to decision. *Bilingualism* 5 175–197. 10.1017/S1366728902003012 15905078

[B46] Donaldson B. (2011). Left-dislocation in near-native French. *Stud. Second Lang. Acquis.* 33 399–432. 10.1017/S0272263111000039

[B47] Dörnyei Z. Skehan P. (2003). “Individual differences in second language learning,” in *The Handbook of Second Language Acquisition* eds Doughty C. J. Long M. H. (Malden, MA: Blackwell), 589–630. 10.1002/9780470756492.ch18

[B48] Elman J. L. Bates E. A. Johnson M. H. Karmiloff-Smith A. Parisi D. Plunkett K. (1996). *Rethinking Innateness: A Connectionist Perspective on Development.* Cambridge, MA: MIT Press.

[B49] Erard M. (2012). *Babel No More: The Search for the World’s Most Extraordinary Language Learners.* New York, NY: Free Press.

[B50] Ettlinger M. Bradlow A. Wong P. C. M. (2014). Variability in the learning of complex morphophonology. *Appl. Psycholinguist.* 35 807–831. 10.1017/S0142716412000586

[B51] Faretta-Stutenberg M. Morgan-Short K. (2018). The interplay of individual differences and context of learning in behavioral and neurocognitive second language development. *Second Lang. Res.* 34 67–101. 10.1177/0267658316684903

[B52] Flege J. E. (1999). “Age of learning and second language speech,” in *Second Language Acquisition and the Critical Period Hypothesis* ed. Birdsong D. (Mahwah, NJ: Lawrence Erlbaum Associates), 101–131.

[B53] Flege J. E. MacKay I. R. A. Piske T. (2002). Assessing bilingual dominance. *Appl. Psycholinguist.* 23 567–598. 10.1017/S0142716402004046

[B54] Flege J. E. Schirru C. MacKay I. R. A. (2003). Interaction between the native and second language phonetic subsystems. *Speech Commun.* 40 467–491. 10.1016/S0167-6393(02)00128-0

[B55] Flege J. E. Yeni-Komshian G. H. Liu S. (1999). Age constraints on‘ second-language acquisition. *J. Mem. Lang.* 41 78–104. 10.1006/jmla.1999.2638

[B56] Foucart A. Frenck-Mestre C. (2012). Can late L2 learners acquire new grammatical features? Evidence from ERPs and eye-tracking. *J. Mem. Lang.* 66 226–248. 10.1016/j.jml.2011/07.007

[B57] Fowler C. A. Sramko V. Ostry D. J. Rowland S. A. Hallé P. (2008). Cross language phonetic influences on the speech of French-English bilinguals. *J. Phon.* 36 649–663. 10.1016/j.wocn.2008.04.001 19802325 PMC2598425

[B58] Garcia-Sierra A. Rivera-Gaxiola M. Peraccio C. R. Conboy B. T. Romo H. Klarman S. O. (2011). Bilingual language learning: an ERP study relating early brain responses to speech, language input, and later word production. *J. Phon.* 39 546–557. 10.1016/j.wocn.2011.07.002

[B59] Goto Butler Y. Hakuta K. (2004). “Bilingualism and second language acquisition,” in *Handbook of Bilingualism* eds Bhatia T. Ritchie W. (New York: Blackwell Publishers), 114–144.

[B60] Granena G. Long M. H. (2013). Age of onset, length of residence, language aptitude, and ultimate L2 attainment in three linguistic domains. *Second Lang. Res.* 29 311–343. 10.1177/0267658312461497

[B61] Green D. W. (2003). “The neural basis of the lexicon and the grammar in L2 acquisition: the convergence hypothesis,” in *The Interface Between Syntax and the Lexicon in Second Language Acquisition* eds van Hout R. Hulk A. Kuiken F. Towell R. (Amsterdam: John Benjamins), 197–218. 10.1075/lald.30.10gre

[B62] Grosjean F. (1989). Neurolinguists, beware! The bilingual is not two monolinguals in one person. *Brain Lang.* 36 3–15. 10.1016/0093-934X(89)90048-5 2465057

[B63] Grosjean F. (2010). *Bilingual: Life and Reality.* Cambridge, MA: Harvard University Press 10.4159/9780674056459

[B64] Hakuta K. Bialystok E. Wiley E. W. (2003). Critical evidence: a test of the critical-period hypothesis for second-language acquisition. *Psychol. Sci.* 14 31–38. 10.1111/1467-9280.01415 12564751

[B65] Hartshorne J. K. Germine L. T. (2015). When does cognitive functioning peak? The asynchronous rise and fall of different cognitive abilities across the life span. *Psychol. Sci.* 26 433–443. 10.1177/0956797614567339 25770099 PMC4441622

[B66] Hartshorne J. K. Tenenbaum J. B. Pinker S. (in press). A critical period for second language acquisition: evidence from 2/3 million English speakers. *Cognition.* 10.1016/j.cognition.2018.04.007 PMC6559801 29729947

[B67] Huang B. H. (2016). A synthesis of empirical research on the linguistic outcomes of early foreign language instruction. *Int. J. Multiling.* 13 257–273. 10.1080/14790718.2015.1066792

[B68] Hurford J. R. (1991). The evolution of the critical period for language acquisition. *Cognition* 40 159–201. 10.1016/0010-0277(91)90024-X 1786674

[B69] Hyltenstam K. (ed.). (2016). *Advanced Proficiency and Exceptional Ability in Second Languages.* Berlin: Mouton de Gruyter 10.1515/9781614515173

[B70] Hyltenstam K. Abrahamsson N. (2003). “Maturational constraints in SLA,” in *The Handbook of Second Language Acquisition* eds Doughty C. J. Long M. H. (Malden, MA: Blackwell), 539–588.

[B71] Indefrey P. (2006). It is time to work toward explicit processing models for native and second language speakers. *Appl. Psycholinguist.* 27 66–69. 10.1017/S0142716406280032

[B72] Johnson J. S. Newport E. L. (1989). Critical period effects in second language learning: the influence of maturational state on the acquisition of English as a Second Language. *Cogn. Psychol.* 21 60–99. 10.1016/0010-0285(89)90003-0 2920538

[B73] Köpke B. Schmid M. S. (2004). “Language attrition: the next phase,” in *First Language Attrition: Interdisciplinary Perspectives on Methodological Issues* eds Schmid M. S. Köpke B. Keijzer M. Weilemar L. (Amsterdam: John Benjamins), 1–43.

[B74] Kovács Á. M. Mehler J. (2009). Cognitive gains in 7-month-old bilingual infants. *Proc. Natl. Acad. Sci. U.S.A.* 106 6556–6560. 10.1073/pnas.0811323106 19365071 PMC2672482

[B75] Lee N. (2004). “The neurobiology of procedural memory,” in *The Neurobiology of Learning: Perspectives from Second Language Acquisition* eds Schumann J. H. Crowell S. E. Jones N. E. Lee N. Schuchert S. A. Wood L. A. (Mahwah, NJ: Lawrence Erlbaum Associates), 43–73.

[B76] Lenneberg E. H. (1967). *Biological Foundations of Language.* New York, NY: Wiley.

[B77] Liu H. Cao F. (2016). L1 and L2 processing in the bilingual brain: a meta-analysis of neuroimaging studies. *Brain Lang.* 159 60–73. 10.1016/j.bandl.2016.05.013 27295606

[B78] Long M. H. (1990). Maturational constraints on language development. *Stud. Second Lang. Acquis.* 12 251–285. 10.1017/S0272263100009165

[B79] Luk G. Bialystok E. (2013). Bilingualism is not a categorical variable: interaction between language proficiency and usage. *J. Cogn. Psychol.* 25 605–621. 10.1080/20445911.2013.759574 PMC3780436 24073327

[B80] Mack M. (1989). Consonant and vowel perception and production: early English-French bilinguals and English monolinguals. *Percept. Psychophys.* 46 187–200. 10.3758/BF03204982 2762107

[B81] MacLeod A. N. Stoel-Gammon C. (2005). Are bilinguals different? What VOT tells us about simultaneous bilinguals. *J. Multicult. Commun. Disord.* 3 118–127. 10.1080/14769670500066313

[B82] MacWhinney B. (2005). “A unified model of language acquisition,” in *Handbook of Bilingualism: Psycholinguistic Approaches* eds Kroll J. F. de Groot A. M. B. (New York, NY: Oxford University Press), 49–67.

[B83] Marchman V. A. (1993). Constraints on plasticity in a connectionist model of the English past tense. *J. Cogn. Neurosci.* 5 215–234. 10.1162/jocn.1993.5.2.215 23972155

[B84] May S. (2014). *The Multilingual Turn: Implications for SLA, TESOL and Bilingual Education.* New York, NY: Routledge.

[B85] Mella N. Fagot D. de Ribaupierre A. (2016). Dispersion in cognitive functioning: age differences over the lifespan. *J. Clin. Exp. Neuropsychol.* 38 111–126. 10.1080/13803395.2015.1089979 26618890

[B86] Meulman N. Wieling M. Sprenger S. A. Stowe L. A. Schmid M. S. (2015). Age effects in L2 grammar processing as revealed by ERPs and how (not) to study them. *PLoS One* 10:e0143328. 10.1371/journal.pone.0143328 26683335 PMC4686163

[B87] Montrul S. (2016a). “Dominance and proficiency in early and late bilingualism,” in *Language Dominance in Bilinguals: Issues of Measurement and Operationalization* eds Silva-Corvalán C. Treffers-Daller J. (Cambridge: Cambridge University Press), 15–35. 10.1017/CBO9781107375345.002

[B88] Montrul S. (2016b). “Age of onset of bilingualism effects and availability of input in first language attrition,” in *Bilingualism Across the Lifespan: Factors Moderating Language Proficiency* eds Nicoladis E. Montanari S. (Washington, DC: American Psychological Association), 141–161.

[B89] Morgan-Short K. Faretta-Stutenberg M. Brill-Schuetz K. A. Carpenter H. Wong P. C. M. (2014). Declarative and procedural memory as individual differences in second language acquisition. *Bilingualism* 17 56–72. 10.1017/S1366728912000715

[B90] Moyer A. (2014). Exceptional outcomes in L2 phonology: the critical factors of learner engagement and self-regulation. *Appl. Linguist.* 34 418–440. 10.1093/applin/amu012

[B91] Muñoz C. Singleton D. (2011). A critical review of age-related research on L2 ultimate attainment. *Lang. Teach.* 44 1–35. 10.1017/S0261444810000327

[B92] Onnis L. Chun W. E. Lou-Magnuson M. (2018). Improved statistical learning abilities in adult bilinguals. *Bilingualism* 21 427–433. 10.1017/S1366728917000529

[B93] Ortega L. (2009). *Understanding Second Language Acquisition.* London: Hodder.

[B94] Pallier C. (2007). “Critical periods in language acquisition and language attrition,” in *Language Attrition: Theoretical Perspectives* eds Köpke B. Schmid M. S. Keijzer M. Dostert S. (Amsterdam: John Benjamins), 155–168. 10.1075/sibil.33.11pal

[B95] Penfield W. Roberts L. (1959). *Speech and Brain Mechanisms.* Princeton, NJ: Princeton University Press.

[B96] Petitto L. A. Berens M. S. Kovelman I. Dubins M. H. Jasinska K. Shalinsky M. (2012). The “perceptual wedge hypothesis” as the basis for bilingual babies’ phonetic processing advantage: new insights from fNIRS brain imaging. *Brain Lang.* 121 130–143. 10.1016/j.bandl.2011.05.003 21724244 PMC3192234

[B97] Pinker S. (1994). *The Language Instinct.* New York, NY: William Morrow 10.1037/e412952005-009

[B98] Pring L. (2007). Savant talent. *Dev. Med. Child Neurol.* 47 500–503. 10.1017/S0012162205000976 15991873

[B99] Prull M. W. Gabrieli J. D. E. Bunge S. A. (1999). “Age-related changes in memory: a cognitive neuroscience perspective,” in *The Handbook of Aging and Cognition* eds Craik F. I. M. Salthouse T. A. (Mahwah, NJ: Lawrence Erlbaum Associates), 91–153.

[B100] Pulvermüller F. Schumann J. H. (1994). Neurobiological mechanisms of language acquisition. *Lang. Learn.* 44 681–734. 10.1111/j.1467-1770.1994.tb00635.x

[B101] Qureshi M. A. (2016). A meta-analysis: age and second language grammar acquisition. *System* 60 147–160. 10.1016/j.system.2016.06.001

[B102] Reichle R. V. Birdsong D. (2014). Processing focus structure in L1 and L2 French: L2 proficiency effects on ERPs. *Stud. Second Lang. Acquis.* 36 535–564. 10.1017/S0272263113000594

[B103] Rossi E. Díaz M. Kroll J. F. Dussias P. E. (2017). Late bilinguals are sensitive to unique aspects of second language processing: evidence from clitic pronouns word-order. *Front. Psychol.* 8:342. 10.3389/fpsyg.2017.00342 28367130 PMC5355469

[B104] Schmeißer A. Hager M. Gil L. A. Jansen V. Geveler J. Eichler N. (2016). “Related but different: the two concepts of language dominance and language proficiency,” in *Language Dominance in Bilinguals: Issues of Measurement and Operationalization* eds Silva-Corvalán C. Treffers-Daller J. (Cambridge: Cambridge University Press), 36–65.

[B105] Schwartz A. I. Kroll J. F. (2006). Bilingual lexical activation in sentence context. *J. Mem. Lang.* 55 197–212. 10.1016/j.jml.2006.03.004

[B106] Scovel T. (1988). *A Time to Speak: A Psycholinguistic Inquiry into the Critical Period for Human Speech.* Rowley, MA: Newbury House.

[B107] Sebastián-Galles N. Díaz B. (2012). First and second language speech perception: graded learning. *Lang. Learn.* 62 (Suppl. 2), 131–147. 10.1111/j.1467-9922.2012.00709.x

[B108] Singleton D. (2017). Language aptitude: desirable trait of acquirable attribute? *Stud. Second Lang. Learn. Teach.* 7 89–103. 10.14746/ssllt.2017.7.1.5

[B109] Smith N. Tsimpli I. Morgan G. Woll B. (2011). *The Signs of a Savant: Language Against the Odds.* Cambridge: Cambridge University Press.

[B110] Steinhauer K. (2014). Event-related potentials (ERPs) in second language research: a brief introduction to the technique, a selected review, and an invitation to reconsider critical periods in L2. *Appl. Linguist.* 35 393–417. 10.1093/applin/amu028

[B111] Sundara M. Polka L. Baum S. (2006). Production of coronal stops by simultaneous bilingual adults. *Bilingualism* 9 97–114. 10.1017/S1366728905002403

[B112] Van Hell J. Abdollahi F. (2017). “Individual variation in syntactic processing in the second language: behavioral and electrophysiological approaches,” in *Developmental Perspectives in Written Language and Literacy* eds Segers E. van den Broek P. (Amsterdam: John Benjamins), 257–273. 10.1075/z.206.16van

[B113] Vanhove J. (2013). The critical period hypothesis in second language acquisition: a statistical critique and a reanalysis. *PLoS One* 8:e69172. 10.1371/journal.pone.0069172 23935947 PMC3723803

[B114] Veríssimo J. Heyer V. Jacob G. Clahsen H. (2017). Selective effects of age of acquisition on morphological priming: evidence for a sensitive period. *Lang. Acquis.* 1–12. 10.1080/10489223.2017.1346104

[B115] Volkow N. D. Gur R. C. Wang G. J. Fowler J. S. Moberg P. J. Ding Y. S. (1998). Association between decline in brain dopamine activity with age and cognitive and motor impairment in healthy individuals. *Am. J. Psychiatry* 155 344–349. 10.ll76/ajp.155.3.344 9501743

[B116] Werker J. F. Hensch T. K. (2015). Critical periods in speech perception: new directions. *Annu. Rev. Psychol.* 66 173–196. 10.1146/annurev-psych-010814-015104 25251488

[B117] Wong P. C. M. Morgan-Short K. Ettlinger M. Zheng J. (2012). Linking neurogenetics and individual differences in language learning: the dopamine hypothesis. *Cortex* 48 1091–1102. 10.1016/j.cortex.2012.03.017 22565204 PMC3965203

[B118] Wong P. C. M. Vuong L. C. Liu K. (2017). Personalized learning: from neurogenetics of behaviors to designing optimal language training. *Neuropsychologia* 98 192–200. 10.1016/j.neuropsychologia.2016.10.002 27720749 PMC5380587

[B119] Yow W. Q. Li X. (2015). Balanced bilingualism and early age of second language acquisition as the underlying mechanisms of a bilingual executive control advantage: why variations in bilingual experiences matter. *Front. Psychol.* 6:164. 10.3389/fpsyg.2015.00164 25767451 PMC4341428

